# Correction: Indocyanine-green-loaded microbubbles for localization of sentinel lymph node using near-infrared fluorescence/ultrasound imaging: a feasibility study

**DOI:** 10.1039/d3ra90075d

**Published:** 2023-08-14

**Authors:** Long Wang, Yihe Hu, Qinghai Peng, Jiawei Zhou, Qichang Zhou, Senbo An, Chengcheng Niu

**Affiliations:** a Department of Ultrasound, The Second Xiangya Hospital, Central South University Changsha 410000 P. R. China cici204675@163.com +86 85292140; b Department of Orthopedics, Xiangya Hospital, Central South University Changsha 410000 P. R. China

## Abstract

Correction for ‘Indocyanine-green-loaded microbubbles for localization of sentinel lymph node using near-infrared fluorescence/ultrasound imaging: a feasibility study’ by Long Wang *et al.*, *RSC Adv.*, 2016, **6**, 50513–50520, https://doi.org/10.1039/C5RA26814A.

The authors regret an error in the original article whereby identical images were presented in Fig. 5a for ICG-PLGA (2 h) and in Fig. 5b for ICG (0 h) and ICG-PLGA (2 h). The correct Fig. 5 is provided here.

**Fig. 1:**
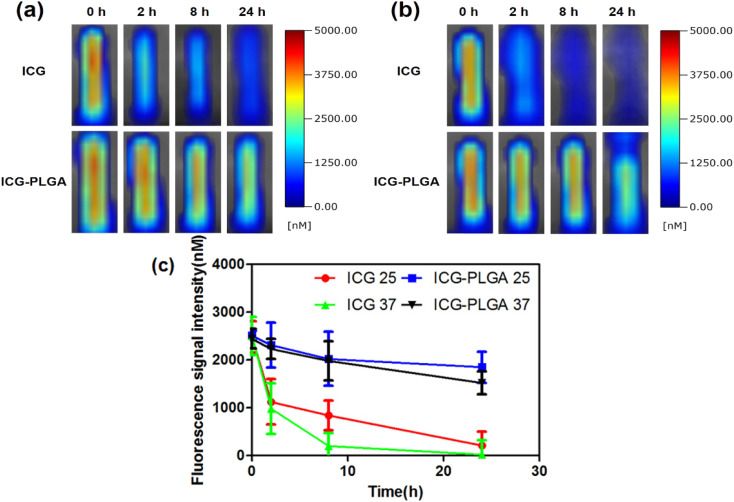
Comparison of the fluorescence stability of ICG and ICG-PLGA microbubbles. Using the NIR fluorescence images, ICG-PLGA microbubbles were compared to ICG in water at (a) 25 °C and (b) 37 °C within a time of 24 hours. Plot for fluorescence stability of the ICG and ICG-PLGA microbubbles over time (24 hours) when incubated in water at 25 °C and 37 °C (c).

The Royal Society of Chemistry apologises for these errors and any consequent inconvenience to authors and readers.

## Supplementary Material

